# Structural comparisons reveal diverse binding modes between nucleosome assembly proteins and histones

**DOI:** 10.1186/s13072-022-00452-9

**Published:** 2022-05-24

**Authors:** Jasmita Gill, Anuj Kumar, Amit Sharma

**Affiliations:** 1 ICMR-National Institute of Malaria Research, New Delhi, India; 2 Molecular Biology Group, ICMR-National Institute of Cancer Prevention and Research, Noida, India; 3 International Centre for Genetic Engineering and Biotechnology, New Delhi, India

**Keywords:** Nucleosome assembly protein, NAP, Histone chaperone, Structural analysis

## Abstract

Nucleosome assembly proteins (NAPs) are histone chaperones that play a central role in facilitating chromatin assembly/disassembly which is of fundamental importance for DNA replication, gene expression regulation, and progression through the cell cycle. In vitro*,* NAPs bind to the core histones H2A, H2B, H3, H4 and possibly to H1. The NAP family contains well-characterized and dedicated histone chaperone domain called the NAP domain, and the NAP–histone interactions are key to deciphering chromatin assembly. Our comparative structural analysis of the three three-dimensional structures of NAPs from *S. cerevisiae*, *C. elegans,* and *A. thaliana* in complex with the histone H2A–H2B dimer reveals distinct and diverse binding of NAPs with histones. The three NAPs employ distinct surfaces for recognizing the H2A–H2B dimer and vice versa. Though histones are highly conserved across species they display diverse footprints on NAPs. Our analysis indicates that understanding of NAPs and their interaction with histone H2A–H2B remains sparse. Due to divergent knowledge from the current structures analyzed here, investigations into the dynamic nature of NAP–histone interactions are warranted.

**Supplementary Information:**

The online version contains supplementary material available at 10.1186/s13072-022-00452-9.

## Background

A nucleosome, the fundamental structural unit of nucleoprotein complexes, is made by the association of DNA with highly basic nuclear proteins called histones. For nucleosome formation, the genomic DNA wraps around a core histone octamer which consists of two molecules of H2A, H2B, H3, and H4 each [1, 2]. This stepwise process that occurs inside the nucleus starts with the formation of H3–H4 dimer followed by oligomerization of H3–H4 tetramer. Subsequently, two H2A–H2B dimers mount onto this tetramer in stepwise-manner to form a histone octamer. A total of 147 bp DNA wraps around this octamer taking nearly two turns and thus completing the production of the nucleosome [1]. Linker histone protein H1 resides on top of the structure keeping the wrapped DNA intact [3]. These nucleosomes perform two main functions, first, they act as a platform for the formation of higher-order chromatin, and second, they affect regulatory control of gene expression [4, 5].

Histone chaperones are the extrinsic factors that promote proper interaction/mounting/unmounting of histone on DNA by shielding the basic histones and thus preventing promiscuous interactions that can lead to cell death. Histone chaperones also modulate the availability of histones and histone variants and orchestrate the mounting of histone to facilitate nucleosome assembly/disassembly [5–8]. By mediating the cross-talk between cellular components, histone chaperones are known to regulate critical cellular pathways of replication, transcription, and DNA repair [3–5, 8, 9]. The essential role played by histone chaperones makes them promising targets for drug development [10]. Several histone chaperones like nucleosome assembly protein (NAP) and others including nucleoplasmin, anti-silencing function (Asf1), chromatin assembly factor (CAF1), and histone regulator A (HirA) are involved in crucial steps of nucleosome/chromatin assembly/disassembly [3, 4, 8, 11, 12].

Nucleosome assembly protein (NAP) family, which is highly conserved across species from unicellular to multicellular organisms, is well-characterized and is a widely studied histone chaperone [5]. NAP is pivotal in the formation, stabilization, and dynamics of chromatin in eukaryotes via several functions. NAP is essential for the formation of histone octamer [13]. The role of NAP in unmounting of histone has also been established during DNA replication, where NAP possibly snatches histone H2A–H2B by taking advantage of structural breathing in the nucleosome, which leads to transitional exposure of histones regions bound to DNA. NAP likely competes with the same binding regions of histones that interact with DNA and gets covered during the wrapping of DNA around nucleosomes [14]. NAP promotes chromatin fluidity by shuttling histones across the nuclear membrane [15]. Interestingly, a proposed role of the NAP family is their likely involvement in nucleosome disassembly rather than nucleosome formation [5, 7]. In this work, we describe the distinct modes of binding of three NAPs from different species when interacting with histone H2A–H2B based on crystal structure analysis of their complexes. We highlight key structural similarities and differences of NAP–histone interactions, and this work emphasizes the need for further analysis of the highly dynamic interactions between NAP and histones.

### The core three-dimensional structure of nucleosome assembly protein

Several three-dimensional apo structures of nucleosome assembly protein (NAPs) from unicellular eukaryotic fungi like *Saccharomyces cerevisiae, Schizosaccharomyces pombe,* and *Pneumocystis carinii* and the malaria parasites *Plasmodium falciparum* and *Plasmodium knowlesi* are available (Protein Data Bank; www.rcsb.org) [1, 10, 16–18]. Apo structures from multicellular organisms *Caenorhabditis elegans*, *Arabidopsis thaliana,* and *Homo sapiens* are also known [19–22]. Three complex structures of histone H2A–H2B dimer bound to NAP are available from *S. cerevisiae* (ScNAP1; PDB ID: 5G2E), *C. elegans* (CeNAP1; PDB IDs: 6K00 and 6K09), and *A. thaliana* (AtNRP1; PDB ID: 7C7X) [19, 20, 23]. Although in vitro binding of NAP1 to all core histones has been reported [11, 12, 24, 25], no structural interpretation is available for NAP1 binding to histones H3 and H4.

**Fig. 1:**
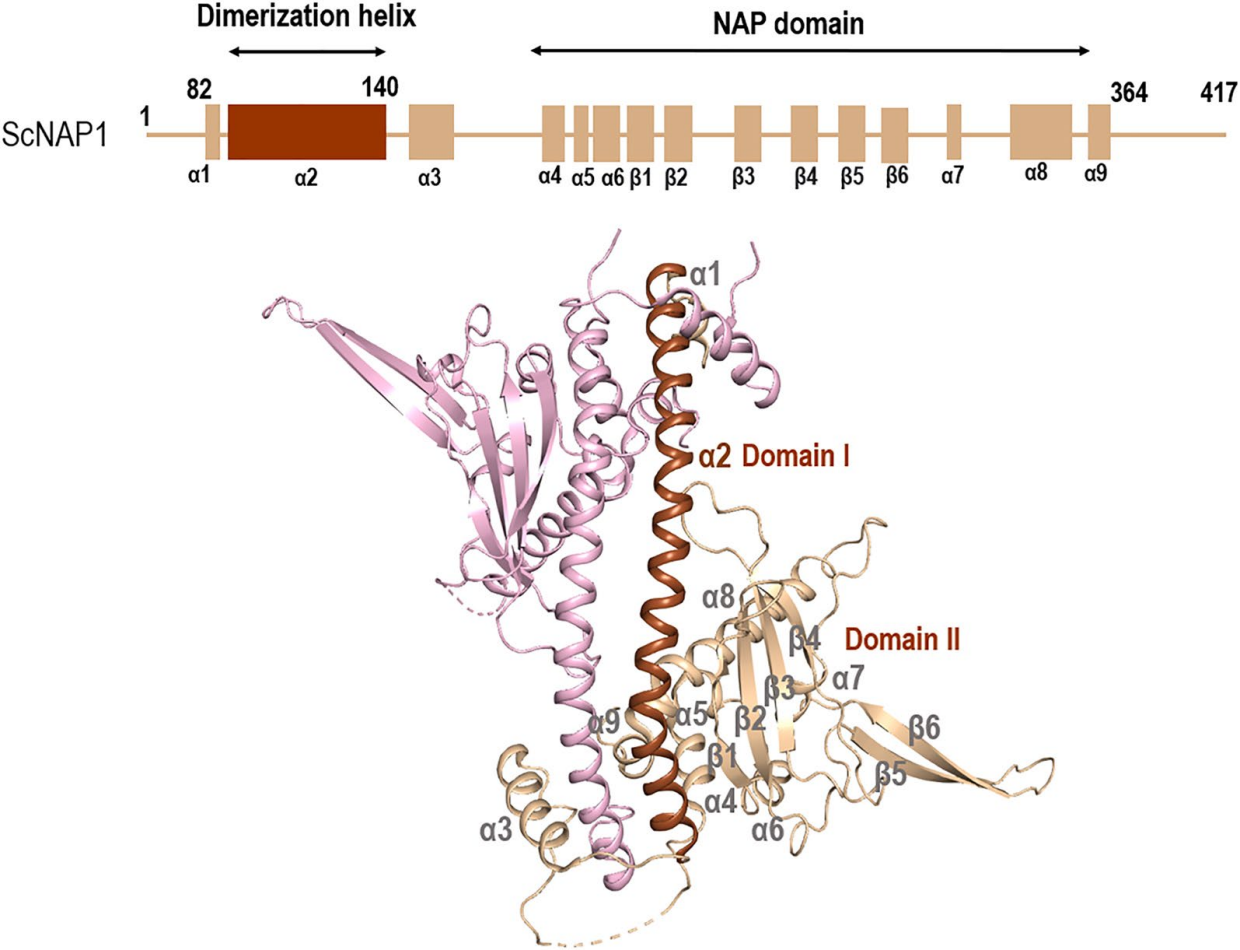
The three-dimensional apo structure of nucleosome assembly protein (NAP) from *S. cerevisiae* (ScNAP1) (PDB ID: 2Z2R). All NAP proteins, including ScNAP1, have the overall NAP fold consisting of a domain I, which is a long dimerization helix (colored brown) and domain II, an “earmuff” NAP domain (colored tan). The other monomer is colored pink. The secondary structure details are collated from PDBSUM (http://www.ebi.ac.uk/thornton-srv/databases/cgi-bin/pdbsum/GetPage.pl?pdbcode=index.html)

NAPs are highly conserved across species and have a dedicated domain known as Nucleosome assembly protein 1-like fold [4]. The overall fold of NAP protein, first reported in *S. cerevisiae* (ScNAP1) in 2006, is seen to be conserved in all NAP structures available (Fig. 1) [1]. This fold consists of a homodimer organized in the form of earmuffs/headphone. The N-terminal and C-terminal acidic domain (CTAD) of ScNAP1 are disordered and are not part of the core structure [1]. The ScNAP1 monomer consists of two domains; domain I acts as the dimerization domain and consists of one short and one long dimerization α-helix (Fig. 1) [1, 15, 26]. Domain II consists of 4 antiparallel β-strands of β-subdomain, which is the signature domain of histone chaperones. The dimerization helix is connected to the β-subdomain by α-helices α3, α4, α5 and α6. This short α3-helix is also known as the accessory domain and is interestingly absent in NAPs from *Plasmodium falciparum* (PfNapL and PfNapS; where L and S stand for long protein and short protein, respectively, based on the protein size) and NAP from *Plasmodium knowlesi* [10]. After the β-subdomain, there are three more α-helices α7, α8, and α9 which connect the β-subdomain with the C-terminal tail. The β-4 sheet and α7 helix are connected by a β-hairpin motif (β-5 and β-6) (Fig. 1).

The NAP family proteins exhibit variation in their nuclear export and localization patterns [10, 17]. The presence of both Nuclear export signal (NES) and Nuclear localization signal (NLS) sequences suggests a possible role of NAP in histone shuttling across the nuclear membrane [1]. NES and NLS are present in ScNAP1 at positions 88-LPKNVKEKLLSLKTLQ-103 and 290-RKQRNK-295 respectively. The accessory domain of NAP regulates the shuttling of ScNAP1 by masking/unmasking the NES sequence [1]. ScNAP1 carries NLS sequence in the β-hairpin motif (β5–β6) [1]. Further, ScNAP1 is localized in the nucleus; however, Drosophila NAP (dNap1) shuttles across the nuclear membrane during cell cycle stages [27]. In embryonic cells, dNap1 localizes in the nucleus during the S phase and moves to the cytoplasm in the G2 phase. *Plasmodium falciparum* PfNapL is localized to the cytoplasm while PfNapS is most likely a resident of the parasite nucleus [10, 17, 24, 25].

### NAP–histone binding modes

#### *Complex 1*: histone H2A–H2B bound to S. cerevisiae NAP1

**Fig. 2:**
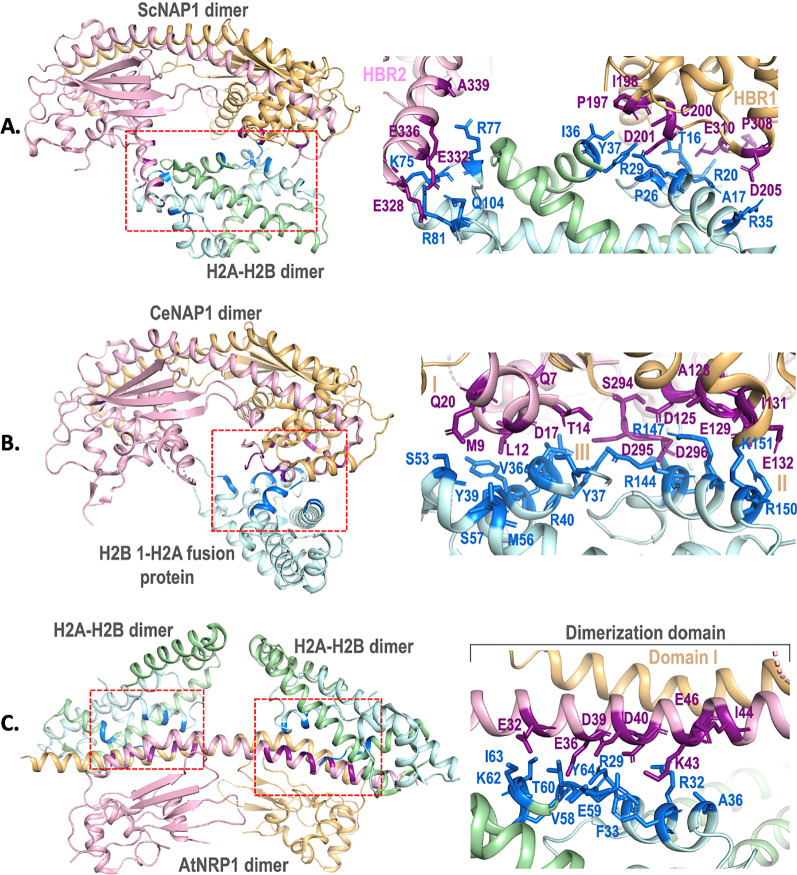
Structural depiction of the histone H2A–H2B dimer interacting residues of ScNAP1, CeNAP1 and AtNRP1. NAP dimer is shown as ribbon and monomers are colored pink and tan. Histones H2A and histone H2B are shown as cyan and green ribbon. The interacting residues of NAPs and histones are shown as violet and blue sticks, respectively. The binding site of histones on each NAP is marked with red boxes. **A** ScNAP1 dimer interacting with H2A–H2B dimer (PDB ID: 5G2E). HBR1, HBR2 and the corresponding interacting residues of ScNAP1 are highlighted. **B** CeNAP1 dimer interacting with H2B 1-H2A fusion protein  (PDB ID: 6K00). Regions I, II, III and the corresponding interacting residues of CeNAP1 are highlighted. **C** AtNRP1 dimer interacting with H2A–H2B dimer (PDB ID: 7C7X). The dimerization domain I and the interacting residues of AtNRP1 are highlighted. Interacting residues are collated from PDBSUM (www.ebi.ac.uk/thornton-srv/databases/cgi-bin/pdbsum/) and all structural depictions are made using Pymol (www.pymol.org)

The low-resolution structure (6.7 Å) of *S. cerevisiae* NAP1 (ScNAP1) in complex with H2A–H2B dimer (from *Xenopus laevis*) shows the involvement of helices α4–α6 of the first NAP monomer and the helix α8 of the second monomer (Fig. 2A, Table 1) suggesting the role of two acidic areas on NAP named histone binding regions - HBR1 and HBR2 (PDB ID: 5G2E). HBR1 spans helices α4–α6 (194–205 amino acids) that interact with α1 of H2A, which is the first DNA region out of three binding regions in histone H2A–H2B dimer [23]. This binding is further consolidated by binding of E310 present at β5–β6 loop to the Nα of H2A. HBR2 at N-terminal of helix α8 (328–336 amino acids) interacts with loops L1 and L2 of H2B and H2A, respectively, which forms the contiguous surface of the second DNA-binding region of histone H2A–H2B dimer. HBR2 (330–356 amino acids) lies in the H–T–H DNA-binding motif of ScNAP1 [23]. The acidic residues are known to interact and stabilize the basic charges of histones (Fig. 2A; Table 1). The residues involved in binding were determined by site-directed mutations [23]. From the HBR1, single, double and triple mutation of D201R, D205R, and D310R of ScNAP1 showed no effect on binding [10, 23]. Similarly, no effect on binding was observed by mutating E332G, D333G, and E336G [10, 23]. However, mutating all the residues to alanine reduced the binding, suggesting a synergistic role of the binding residues [10, 23]. The overall stoichiometry of binding was one ScNAP1 dimer binding to a single H2A–H2B dimer (1:1), which was different from the previously reported stoichiometry of 1:2 [29]. The binding of ScNAP1 to histone H2A–H2B as well as histone variant H2A.Z–H2B has been reported in vivo [1, 26, 30]*.* Furthermore, the deletion of ScNAP1 resulted in reduced deposition of the second H2A–H2B dimer required for histone octamer formation [23].

**Table 1:** Interacting residues of NAP–histone complexes

	Histone-binding residues of NAP	Histone H2A residues that bind to NAP	Histone H2B residues that bind to NAP
		*Xenopus laevis* H2A–H2B	
ScNAP1	P197, I198, C200, D201, D205, P308, E310, E328, E332, E336, A339	T16, R17, R20, P26, R29, R35, K75, R77, R81, Q105	I36, Y37
		*C. elegans* H2B 1–H2A fusion protein	
CeNAP1	Q7, M9, L12, T14, D17, Q20, D125, A128, E129, I131, E132, S294, D295, D296	*S131, R132, G143, R144, R147, R150, K151*	*S33, S35, V36, Y37, Y39, R40, S53, M56, S57*
		A.*thaliana* H2A–H2B	
AtNRP1	E32, E36, D39, D40, K43, I44, E46	R29, R32, F33, A36	V58, E59, T60, K62, I63, Y64

Histone-binding residues of NAPs and NAP-binding residues of histone H2A–H2B based on the three three-dimensional structures of NAP–histone complex—*S*. *cerevisiae* (ScNAP1; PDB ID: 5G2E), *C. elegans* (CeNAP1; PDB ID: 6K00) and *A. thaliana* (AtNRP1; PDB ID: 7C7X). Italicized residues belong to fusion protein H2B 1–H2A

#### *Complex 2*: histone H2A–H2B bound to C. elegans NAP1

**Fig. 3:**
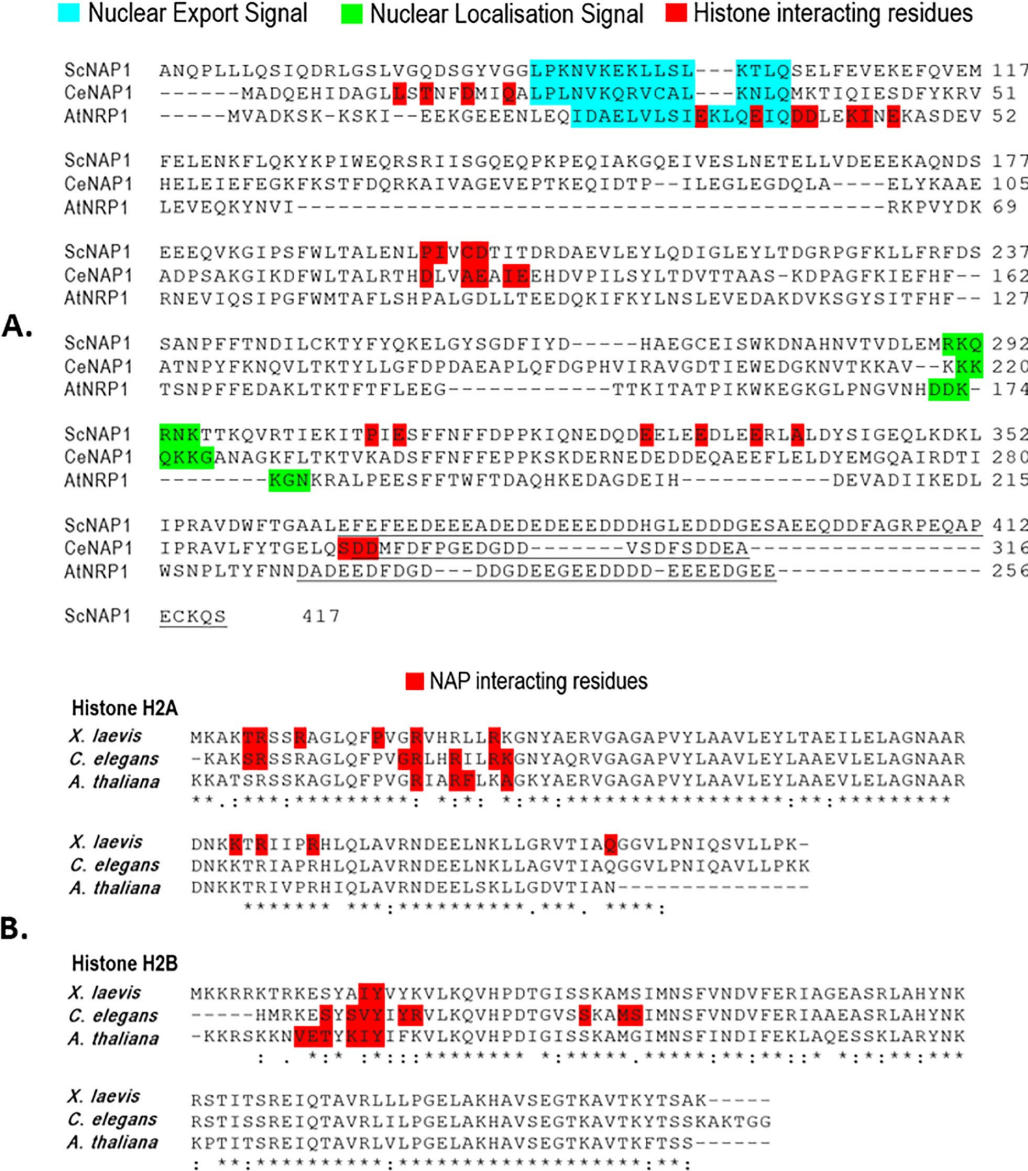
Sequence alignment of NAPs and histones. **A** Alignment of ScNAP1, CeNAP1 and AtNRP1. NES and NLS are colored cyan and green, respectively. The residues of CTAD are underlined. Histone-binding residues are colored red. **B** Alignment of histone H2A and H2B from *X. laevis* (ScNAP1 complex), *C. elegans* and *A. thaliana*. NAP-binding residues of these histones are colored red. Sequences are taken from Protein Data Bank (www.rcsb.org) and sequence alignments are done using Clustal Omega (www.ebi.ac.uk/Tools/msa/clustalo/)

The three-dimensional structures of *C. elegans* NAP1 (CeNAP1) in complex with H2B 1–H2A fusion protein (PDB ID: 6K00 and 6K09) and CeNAP1–H2AZ–H2B complex (PDB ID: 6K0C) are available [20]. CeNAP1 uses three acidic “strips” or regions to interact with H2A–H2B dimer—region I, II, and III (Fig. 2B; Table 1). Region I lies at the N-terminal. Three histone-interacting residues of CeNAP1 lie in the CTAD region (Fig. 3). On mutating the acidic residues of these regions, only region II (D125, E129, E132) and region III (D295, D296) differed in binding with histones. The stoichiometry of binding in CeNAP1 and ScNAP1 is the same, i.e., 1:1; however, unlike ScNAP1, only one monomer of CeNAP1 dimer interacts with the histone dimer. The distribution of acidic residues also leads to a vertical orientation of histone dimers. In addition, CeNAP1showed binding to H3–H4 dimer at the same acidic strip [20].

#### *Complex 3*: histone H2A–H2B bound to NRP1 from A. thaliana

**Fig. 4:**
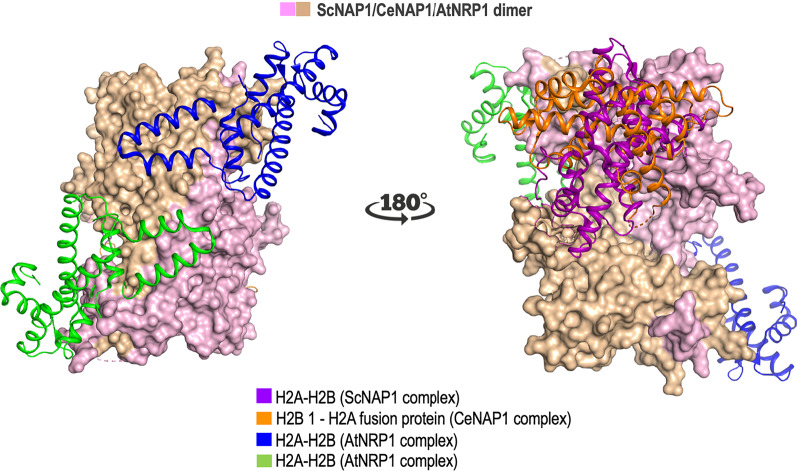
Structural superposition of the three NAP–histone complexes - ScNAP1, CeNAP1 and AtNRP1. Only ScNAP1 dimer is shown as surface in two orientations for simplicity. The monomers are colored pink and tan. Histone H2A–H2B dimer that binds to ScNAP1 is shown as purple ribbon. The histone H2B 1–H2A fusion protein bound at CeNAP1–histone binding region is shown as orange ribbon. Similarly, two H2A–H2B dimers bound at AtNRP1 binding region are shown as blue and green ribbon

The three-dimensional structure of *A. thaliana* NAP1–Related Protein 1 (AtNRP1) in complex with two H2A–H2B dimers (PDB ID: 7C7X) shows a highly distinct mode of AtNRP1 binding to H2A–H2B dimer [19] as the Nα helix of dimerization domain (domain I) of AtNRP1 binds to helices α1 of H2A–H2B (Fig. 2C, Table 1). This binding mode of AtNRP1 is unique when compared to ScNAP1 and CeNAP1. The stoichiometry of binding of AtNRP1 also differs since one AtNRP1 dimer binds to two heterodimers of H2A–H2B, whereas one dimer of ScNAP1 and CeNAP1 binds to only one heterodimer (Fig. 2). Further, AtNRP1 contains an acidic stretch consisting of Asp and Glu residues (E32, E36, D39, D40 and E46) in domain I which is solely involved in the binding to each of the H2A–H2B dimers. The involvement of dimerization helix in the case of AtNRP1 likely supports simultaneous interaction with two H2A–H2B dimers (Figs. 2C, 3, 4, Additional file 1: Figure S1) [19]. Intriguingly, cross-linking experiment and ITC data on binding studies suggest residues from two regions of earmuff domain, K115/D116/D214 (located in the loop L1 connecting β-1 and β-2) and E213/D214 (exists at the terminus of helix α5), are also involved in binding to histone [19]. Additionally, the complex structure of CTAD from *A. thaliana* and CTAD from human NAP1 showed a conversed binding mode to H2A–H2B dimer [19, 31].

### Complexes 1, 2, and 3 reveal distinct modes of interaction between NAPs and histones

Our analysis shows that ScNAP1, CeNAP1, and AtNRP1 share ~ 35% sequence similarity but have similar overall structures. Sequence alignment of these NAPs shows relatively conserved NES and NLS sequences (Fig. 3). Complexes 1, 2, and 3 contain the NAP domain, however, there are structural differences between them. For example, earmuff domain of complex 3 is shorter in comparison to complexes 1 and 2 (Figs. 2, 3). In addition, the loop connecting the dimerization domain to the earmuff domain in complex 3 is longer than that of complexes 1 and 2 (Fig. 2). The angle between the two earmuff domains differs in all three NAPs in apo as well as histone-bound structures [20]. The root mean square deviation (r.m.s.d.) of NAP dimers from complex 1 (504 Cα) and complex 2 (526 Cα) is 2.3 Å and the dimerization helices and earmuff domains superimpose well. The NAP dimers in complex 1 (504 Cα) and complex 3 (348 Cα) exhibit slight deviation between their dimerization helices and the earmuff domains as the r.m.s.d. is 3.4 Å. The deviations in these two domains are most prominent between complex 2 (526 Cα) and complex 3 (348 Cα) with a high r.m.s.d. of 6.5 Å. This could be explained by how two histone H2A–H2B dimers are bound in complex 3 compared to only one in complex 1 (Fig. 4, Additional file 1: Figure S1). Further, in all three complexes, histones utilize their basic residues to mount on NAPs at the NAP acidic regions (Table 1, Figs. 2, 3). Thus, similar types of electrostatic interactions are observed in all three complexes. The C-terminal acidic domain (CTAD) is reported to be involved in histone binding [19, 20, 23], though, intriguingly, these acidic regions are not conserved across species. Most importantly, the manner of binding of histones in all three complexes is considerably different. The acidic region in complexes 1 and 2 is spread across different α-helices that juxtapose to form a single contiguous surface on the earmuff domain (Figs. 2, 4, Additional file 1: Figure S1) [19]. In complex 1, histone interacts with both the monomers of the ScNAP1 dimer since it interacts with the helices α4–α6 of the first monomer and the helix α8 of the second monomer (Fig. 2A). In complex 2, though a similar interaction of CeNAP1 earmuff domain is seen as in complex 1, histone interacts with only one monomer of CeNAP1 (Fig. 2B). Displaying a contrarian position, in complex 3, histone binds on a continuous acidic region on the dimerization helix that is at a 180° turn of the binding regions shown in complexes 1 and 2 (Figs. 2C, 4, Additional file 1: Figure S1). Also, the acidic stretch of domain I seen in complex 3 which is involved in interaction with histone is not conserved in complexes 1 and 2 (Figs. 2C, 3) [19, 20]. Interestingly, there is no involvement of domain II in binding in complex 3 unlike complexes 1 and 2 which share overlapping regions with each other in the earmuff domain II (Figs. 2, 4, Additional file 1: Figure S1). Further, the antiparallel β-sheet domain, the signature domain of NAPs, is not involved in histone binding in all three NAP–histone complexes (Figs. 2, 4). The histone-interacting residues of NAP are varied in complexes 1, 2, and 3 elucidating the remarkable diversity by which NAPs are capable of making interactions with histones (Figs. 2, 4, Additional file 1: Figure S1, Table 1).

#### Histone H2A–H2B footprint on NAPs

**Fig. 5:**
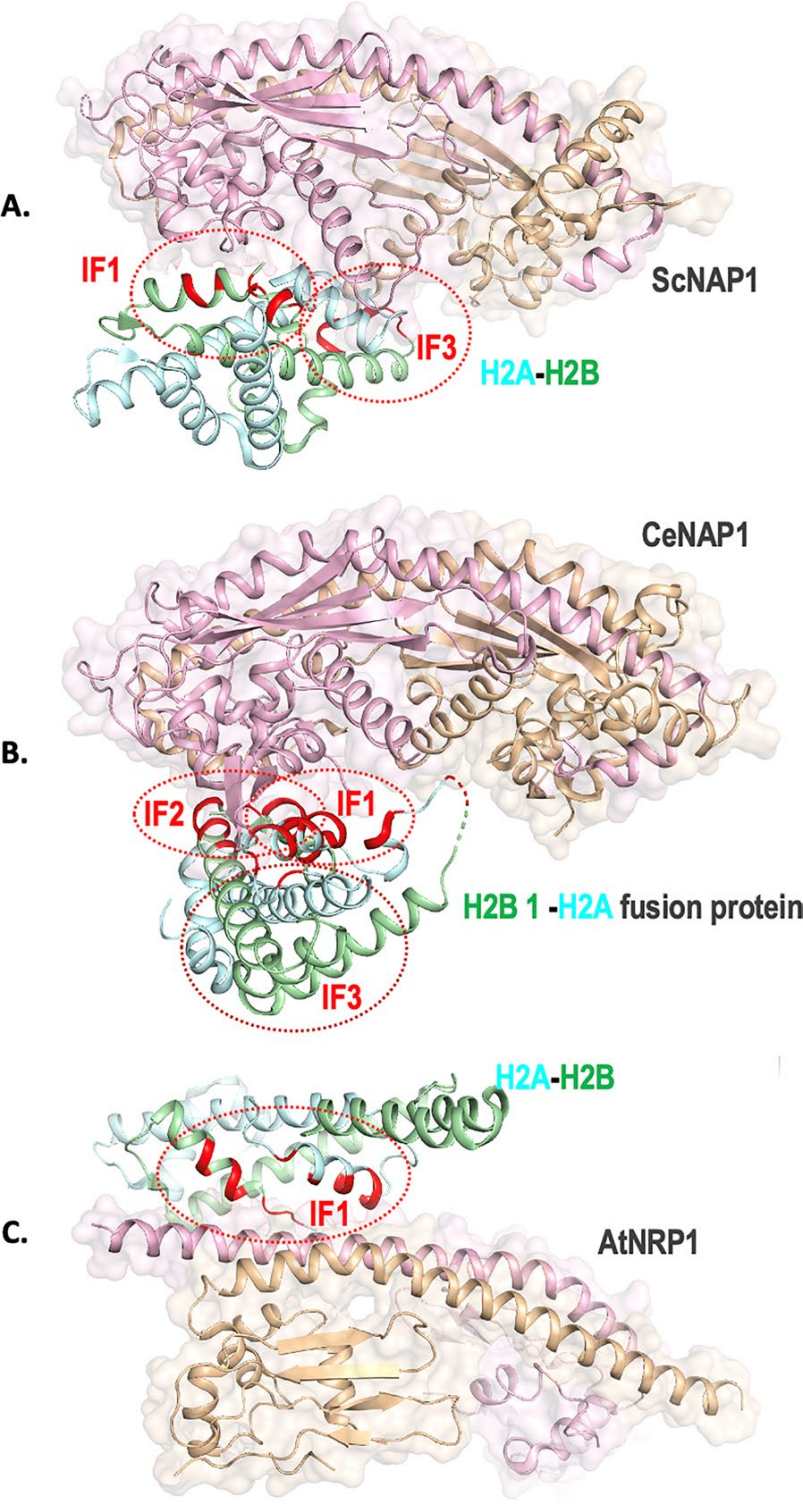
Histone H2A–H2B dimer interaction interfaces (IF) on NAPs. NAP dimers are shown as transparent surface with monomers as pink and tan ribbons. Histone H2A and H2B are shown in cyan and green, respectively. The three interaction interfaces (IF1, IF2 and IF3) of histones are marked with red spheres and the interacting residues of histones that lie in each interface are colored red. **A** ScNAP1-(H2A–H2B) complex. **B** CeNAP1-(H2B 1-H2A) complex. **C** AtNRP1-(H2A–H2B) complex. Only one H2A–H2B dimer is shown for simplicity

Since NAPs transfer the histone H2A–H2B dimers onto the nucleosomes, their binding is of crucial interest to understanding whether the binding footprint on histones overlaps with the DNA-binding regions. It is well-established that histone proteins are conserved across species. The sequence alignment of histones H2A and H2B from complexes 1, 2, and 3 (*X. laevis*,* C. elegans*, and *A. thaliana*, respectively) highlights high sequence similarity between the histones (Fig. 3B). Interestingly, NAP-binding residues of histones in all three complexes are overlapping and conserved (Fig. 3B). However, differences exist in the histone footprint on NAPs, for example, *X. laevis* H2A–H2B dimer in complex 1 uses a greater number of residues compared to complexes 2 and 3 to interact with ScNAP1 (Figs. 2A, 3). Remarkably, though complex 3 employs a completely contrasting mode of interaction between histone and NAP, the histone residues involved in interacting with NAP are still overlapping and conserved with the histones in complex 1 and 2 (Figs. 2, 3). Interestingly, complex 3 also contains two histone H2A–H2B dimers bound to the NAP, unlike complexes 1 and 2 which have only one (Fig. 4). Histones contain histone fold domains, constituting three helices α1, α2, and α3 connected by loops L1 and L2. The histone dimer contains three DNA-binding interfaces (IF) where IF1 consists of H2A L1 and H2B L2; IF2 contains the middle of H2A–H2B (including helices αN and α1 of H2A); IF3 contain L2 of H2A and L1 of H2B and both the termini of the helix α2 (Fig. 5) [32]. As seen in complex 1, the Nα and L2 loop region of H2A and N-terminal of helix α2 binds to ScNAP1 and are not accessible for nucleosome formation (Figs. 2A, 5). This indicates that ScNAP1 binds at IF2 and IF3 of H2A–H2B dimer but has no interaction with IF1 and thus ScNAP1 does not completely overlap with the DNA-binding region. This is in agreement with the results of the histone–peptide interaction experiment of PfNapS [28]. Unlike ScNAP1, only one earmuff domain of CeNAP1 binds to histone covering the α1 and L1 loop region of H2A, and it does not interact with the L2 loop region. The CeNAP1 also interacts with the α1 region of H2B (Fig. 5). Moreover, CeNAP1 binds to the positively charged surface of both H2A and H2B monomers whereas ScNAP1 binds to only H2A. It has been proposed that this variation in binding is due to the multicellularity of *C. elegans* in comparison to yeast which is a unicellular organism [20]. In complex 3, the H2A–H2B dimer uses IF1 to interact with the αN dimerization domain of AtNRP1, whereas the CTAD domain only uses the IF3 of H2A–H2B dimer. The biochemical data suggest that the earmuff domain uses IF2 of H2A–H2B dimer [20, 33].

Our analysis of three-dimensional structures of other histone chaperones complexed with histones suggests that the total binding interface area of NAP with histone H2A–H2B was only half when compared to other histone chaperones like Asf1, HJURP, SME3, DAXX, SPT2, and YL1 [34]. It is likely that the mechanism of binding of NAP to histones is different in comparison to other histone chaperones [19, 20].

## Conclusions

Nucleosome assembly proteins (NAP) are histone chaperones that play an integral part in the production and maintenance of eukaryotic chromatin by facilitating nucleosome assembly/ disassembly. This chromatin assembly is critical for DNA replication, gene expression control, and cell cycle progression. NAPs interact with histone nuclear proteins H2A, H2B, H3, H4, and H1 to mount them onto DNA inside the nucleus. Our analysis of three three-dimensional structures of the NAP–histone complexes reveals distinct modes of histone H2A–H2B recognition by NAPs. Histones also exhibit varied footprints on NAPs. Though NAPs interact with the DNA-binding region of histone H2A and H2B, however, this binding is not consistent in the three complexes. Due to divergence in current complexes in terms of binding modes between NAPs and histones, it is paramount to continue investigations on the overarching mechanism of binding and interaction between NAPs and histones.

## Supplementary Information

**Additional file: Figure S1: **The NAP-histone complexes of ScNAP1, CeNAP1 and AtNRP1. The NAP dimers are shown as surfaces in two orientations. The NAP monomers are colored pink and tan. **A** ScNAP1; histone H2A–H2B dimer is shown as purple ribbon, **B** CeNAP1; the histone H2B 1-H2A fusion protein is shown as orange ribbon, and **C** AtNRP1; the two H2A–H2B dimers bound are shown as blue and green ribbon.

## Data Availability

Not applicable.
