# Supplementary material for: Structural comparisons reveal diverse binding modes between nucleosome assembly proteins and histones
Source: Epigenetics Chromatin. 2022 May 24;15:20. doi: 10.1186/s13072-022-00452-9 (PMC9128123; doi:10.1186/s13072-022-00452-9)
Supplement: Supplementary file 1 — Additional file: Figure S1: The NAP-histone complexes of ScNAP1, CeNAP1 and AtNRP1. The NAP dimers are shown as surfaces in two orientations. The NAP monomers are colored pink and tan. A ScNAP1; histone H2A–H2B dimer is shown as purple ribbon, B CeNAP1; the histone H2B 1-H2A fusion protein is shown as orange ribbon, and C AtNRP1; the two H2A–H2B dimers bound are shown as blue and green ribbon. [file 13072_2022_452_MOESM1_ESM.docx]

**
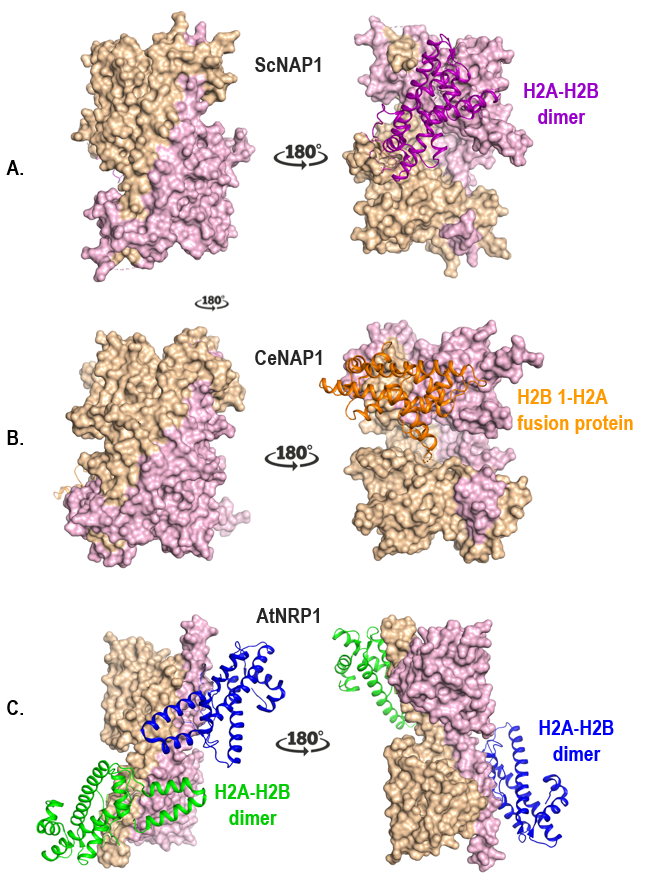
 Figure S1: The NAP-histone complexes of ScNAP1, CeNAP1 and AtNRP1.** The NAP dimer is shown in two orientations as surface. The NAP monomers are coloured pink and tan. The NAP dimers are shown in two orientations. A. ScNAP1; histone H2A-H2B dimer is shown as purple ribbon, B. CeNAP1; the histone H2A-H2B fusion protein is shown as orange ribbon, and C. AtNRP1; the two H2A-H2B dimers bound are shown as blue and green ribbon.
